# A New Tetradentate Mixed Aza-Thioether Macrocycle and Its Complexation Behavior towards Fe(II), Ni(II) and Cu(II) Ions

**DOI:** 10.3390/molecules25092030

**Published:** 2020-04-27

**Authors:** Sze-Wing Ng, Siu-Chung Chan, Chi-Fung Yeung, Shek-Man Yiu, Chun-Yuen Wong

**Affiliations:** 1 Department of Chemistry, City University of Hong Kong, Tat Chee Avenue, Kowloon, Hong Kong; 2 State Key Laboratory of Terahertz and Millimeter Waves, City University of Hong Kong, Tat Chee Avenue, Kowloon, Hong Kong

**Keywords:** first-row transition metal ion, macrocyclic ligand, coordination complex

## Abstract

A new tetradentate mixed aza-thioether macrocyclic ligand 2,6-dithia[7](2,9)-1,10-phenanthrolinophane ([13]ane(phenN_2_)S_2_) was successfully synthesized. Reacting metal precursors [Fe(CH_3_CN)_2_(OTf)_2_], Ni(ClO_4_)_2_·6H_2_O, and Cu(ClO_4_)_2_·6H_2_O with one equivalent of [13]ane(phenN_2_)S_2_ afforded [Fe([13]ane(phenN_2_)S_2_)(OTf)_2_] (**1**), [Ni([13]ane(phenN_2_)S_2_)](ClO_4_)_2_ (**2**(ClO_4_)_2_), and [Cu([13]ane(phenN_2_)S_2_)(OH_2_)](ClO_4_)_2_ (**3**(ClO_4_)_2_), respectively. The structures of [13]ane(phenN_2_)S_2_ and all of its metal complexes were investigated by X-ray crystallography. The [13]ane(phenN_2_)S_2_ was found to behave as a tetradentate ligand via its donor atoms N and S.

## 1. Introduction

Coordination chemistry of first-row transition metal ions has attracted considerable attention for decades due to their high biological relevance. Not only do the coordination complexes serve as models to study biologically related metallomolecules, but they also provide insights into biomimetic designs [1,2,3,4,5,6,7,8,9,10,11,12,13,14,15]. Among a reservoir of ligand designs, macrocycles have been demonstrated to be a valuable type of auxiliary ligand for these studies because (1) many macrocycles are incorporated in naturally-occurring metalloproteins and metalloenzymes, such as porphyrin in haemoglobin and corrin in vitamin B_12_, and (2) the properties of the derived metal complexes can be modulated via modification in the ring size and donor atoms on the macrocyclic ligands.

**Scheme 1:**
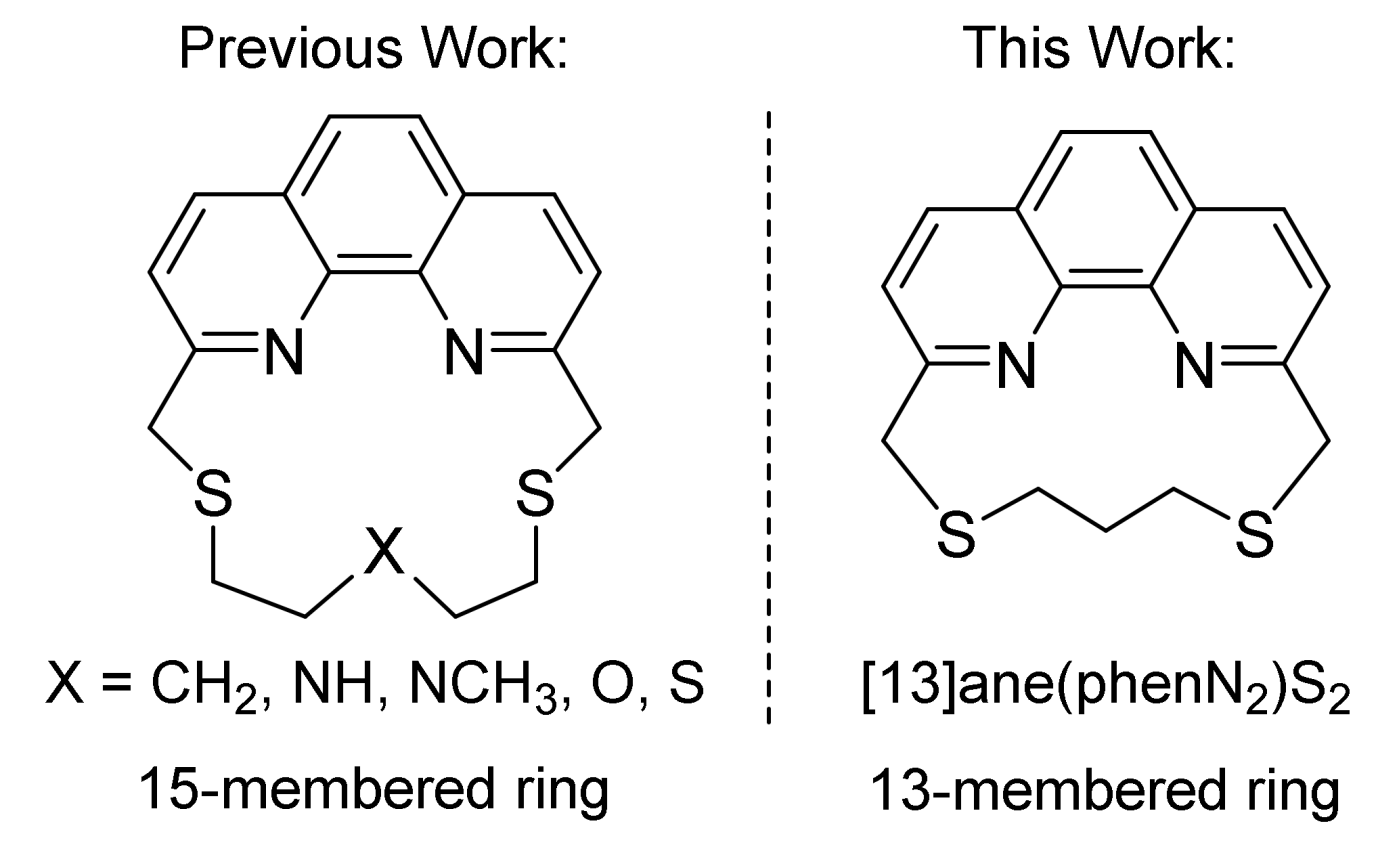
Mixed phenanthroline-thioether macrocycles reported in previous works and this work.

Our group has recently initiated a paradigm in scrutinizing the reactivity between low-valent transition metal precursors and heteroatom-functionalized alkynes [16,17,18,19,20,21,22,23,24,25,26,27,28,29]. Gratifyingly, several Ru- and Os-heterocyclic complexes reported by our group exhibit interesting biological applications. The success of preparing these functional metalated heterocyclic complexes prompted us to expand the scope of our studies to other biologically relevant first-row transition metals. Moreover, we envision that employing multidentate macrocycles as auxiliary ligands in these studies can reduce the ambiguity in mechanistic elucidations because well-designed macrocycles can effectively minimize partial ligand dissociation. With these considerations in mind, we would like to prepare some new first-row transition metal complexes supported by macrocycles for analogous reactivity studies. Among a variety of macrocycles, we are interested in mixed phenanthroline-thioether ligands, not only because their preparation is straightforward but they are well-known to stabilize various low-valent first-row transition metal ions (Scheme 1) [30,31,32,33,34,35,36,37,38,39,40,41,42,43,44,45,46,47]. While most of the reported mixed phenanthroline-thioether macrocycles are a 15-membered ring with 5 donor atoms to behave as pentadentate ligands, analogs with smaller ring sizes that can increase their conformational rigidity are rare. In this context, we report the preparation and complexation ability of a new tetradentate macrocyclic ligand 2,6-dithia [7](2,9)-1,10-phenanthrolinophane (denoted as [13]ane(phenN_2_)S_2_). To our delight, this macrocycle allows the preparation of low-valent transition metal complexes [Fe([13]ane(phenN_2_)S_2_)(OTf)_2_], [Ni([13]ane(phenN_2_)S_2_)](ClO_4_)_2_ and [Cu([13]ane(phenN_2_)S_2_)(OH_2_)](ClO_4_)_2_ under mild reaction conditions with high yields. The solid-state structures of [13]ane(phenN_2_)S_2_ and its metal complexes have been determined by X-ray crystallography. The success of preparing these macrocyclic complexes undoubtedly paves the way for performing further reactivity studies.

## 2. Results and Discussion

### 2.1. Synthesis and Characterization of [13]ane(phenN_2_)S_2_

**Scheme 2:**
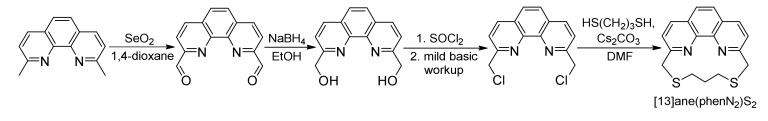
Synthesis of [13]ane(phenN_2_)S_2_.

The new mixed aza-thioether macrocyclic ligand [13]ane(phenN_2_)S_2_ was prepared from cyclization between 2,9-bis(chloromethyl)-1,10-phenanthroline and 1,3-propanedithiol in the presence of Cs_2_CO_3_ as a base and source of template cation under high dilution conditions (Scheme 2). The 2,9-bis(chloromethyl)-1,10-phenanthroline can be synthesized in accordance with previously reported literature methods from 2,9-dimethyl-1,l0-phenanthroline as depicted in Scheme 2 [48,49,50,51,52]. The formation of this air-stable macrocycle was characterized by mass spectroscopy together with ^1^H and ^13^C-NMR spectroscopy.

**Figure 1:**
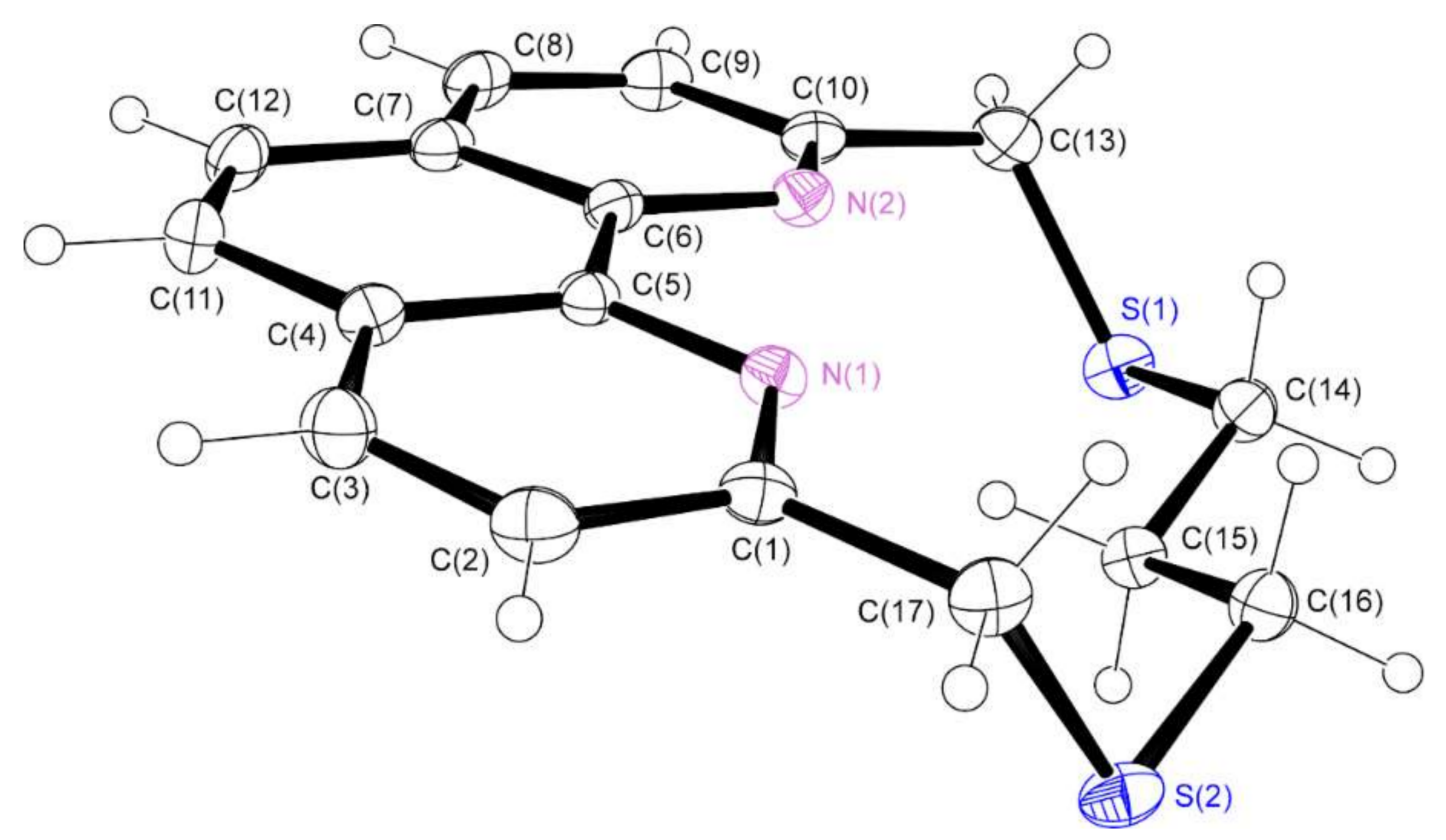
Perspective view of [13]ane(phenN_2_)S_2_ as represented by 30% probability ellipsoids. Selected bond lengths (Å) and angles (°): N(1)-C(1), 1.326(2); N(1)-C(5), 1.352(2); C(5)-C(6), 1.456(2); N(2)-C(6), 1.349(3); N(2)-C(10), 1.323(2); C(1)-C(17), 1.498(3); C(10)-C(13), 1.501(3); S(1)-C(13), 1.822(2); S(1)-C(14), 1.823(2); S(2)-C(16), 1.819(2); S(2)-C(17), 1.822(2); C(1)-C(17)-S(2), 114.6(1), C(10)-C(13)-S(1), 115.3(1); C(13)-S(1)-C(14), 103.5(1); C(16)-S(2)-C(17), 103.8(1).

The structure of [13]ane(phenN_2_)S_2_ was further confirmed by X-ray crystallography (Figure 1). While the phenanthroline moiety of this macrocycle is essentially planar, the 13-membered ring adopts a folded conformation in the solid state. More specifically, the dimethylphenanthroline moiety is essentially planar whereas the -S(CH_2_)_3_S- chain is tilted over the phenanthroline unit, with an angle of 51.6° between the mean planes constructed by C(1), C(10), C(13), C(17) and C(13), C(17), S(1), S(2). The C-C bonds along the -S(CH_2_)_3_S- unit take on *anti* conformations. All the C-S bond distances (1.822(2)-1.823(2) Å) are typical for thioethers [47]. Since the lone pairs of the S-donors adopt exodentate orientation pointing out of the ring cavity, large conformational change is expected for [13]ane(phenN_2_)S_2_ to act as a tetradentate ligand via donor atoms N and S.

**Figure 2:**
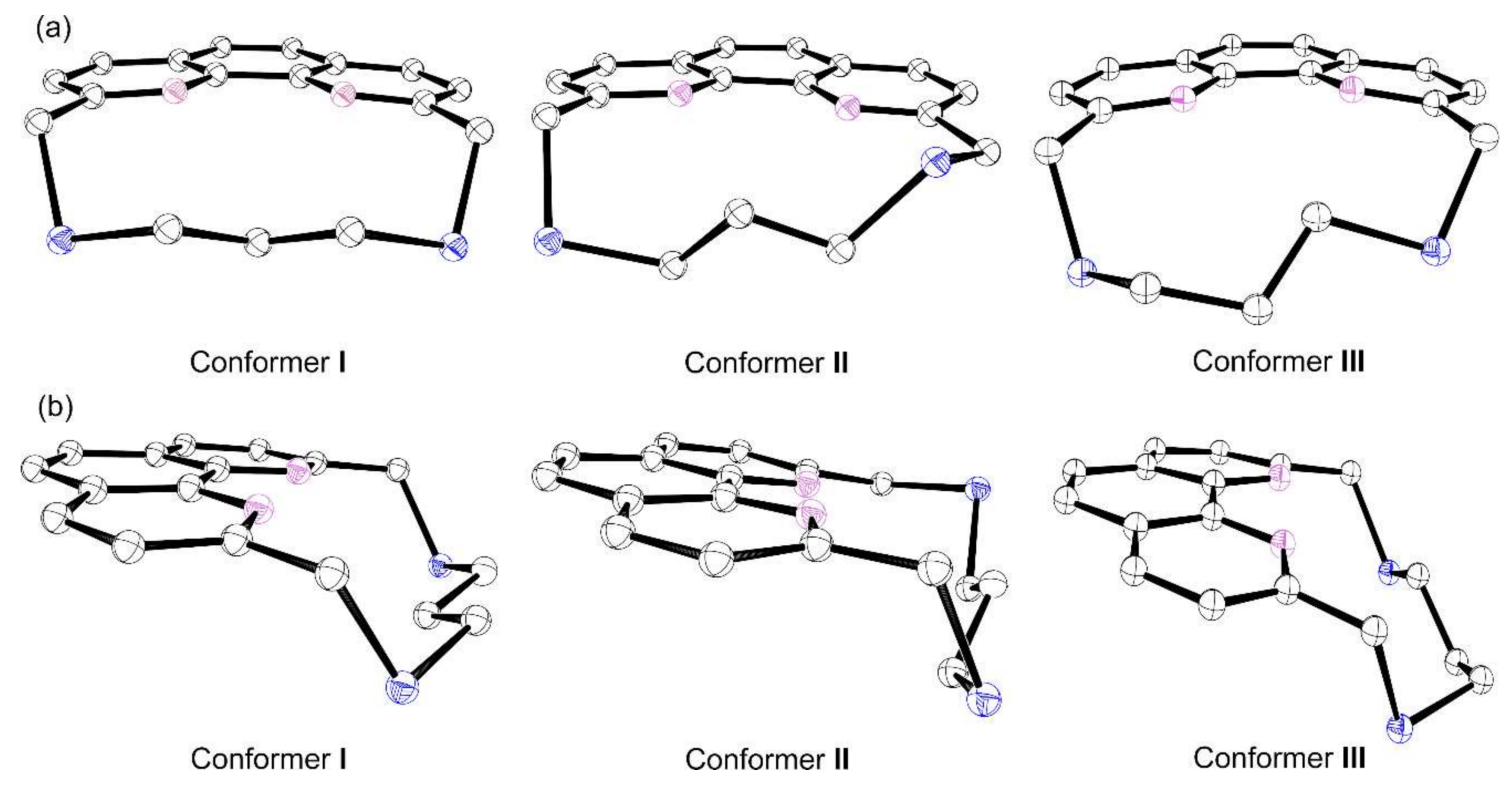
(**a**) Front views and (**b**) side views of three possible low-energy conformers of [13]ane(phenN_2_)S_2_.

To explore the possible low-energy conformers, the X-ray structure for [13]ane(phenN_2_)S_2_ was optimized at the density functional theory (DFT) level to yield the first conformer, Conformer **I**. The search for other low-energy conformers was then performed by exploring the torsional energy surface of the aliphatic chain, followed by structural optimization. Two other conformers, denoted as Conformers **II** and **III**, were obtained throughout the conformer search. These conformers **I**–**III** are labeled in ascending order of their relative energy (0.0, 1.2 and 4.4 kcal mol^−1^, respectively). Their structures are depicted in Figure 2, with selected bond lengths (Å) and angles (°) summarized in Table 1. Notably, the structure of Conformer **I** is in good agreement with the X-ray structure, in which the two S atoms are tilted over the dimethylphenanthroline moiety in the same direction and the two C-C bonds on the -S(CH_2_)_3_S- unit take on *anti* conformation. On the other hand, one of the S atoms in Conformer **II** is coplanar with the dimethylphenanthroline moiety, while another S atom keeps tilting over the phenanthroline unit. Although the two S atoms in Conformer **III** are tilted over the dimethylphenanthroline moiety in the same direction, the C-C bonds on the -S(CH_2_)_3_S- unit take on a combination of *anti* and *gauche* conformations, making it structurally distinct from Conformer **I**. As none of these conformers have their donor atoms pointing to a common center, large distortion is expected for the ligand to act as a tetradentate ligand.

### 2.2. Synthesis and Characterization of [13]ane(phenN_2_)S_2_-Containing Transition Metal Complexes

**Scheme 3:**
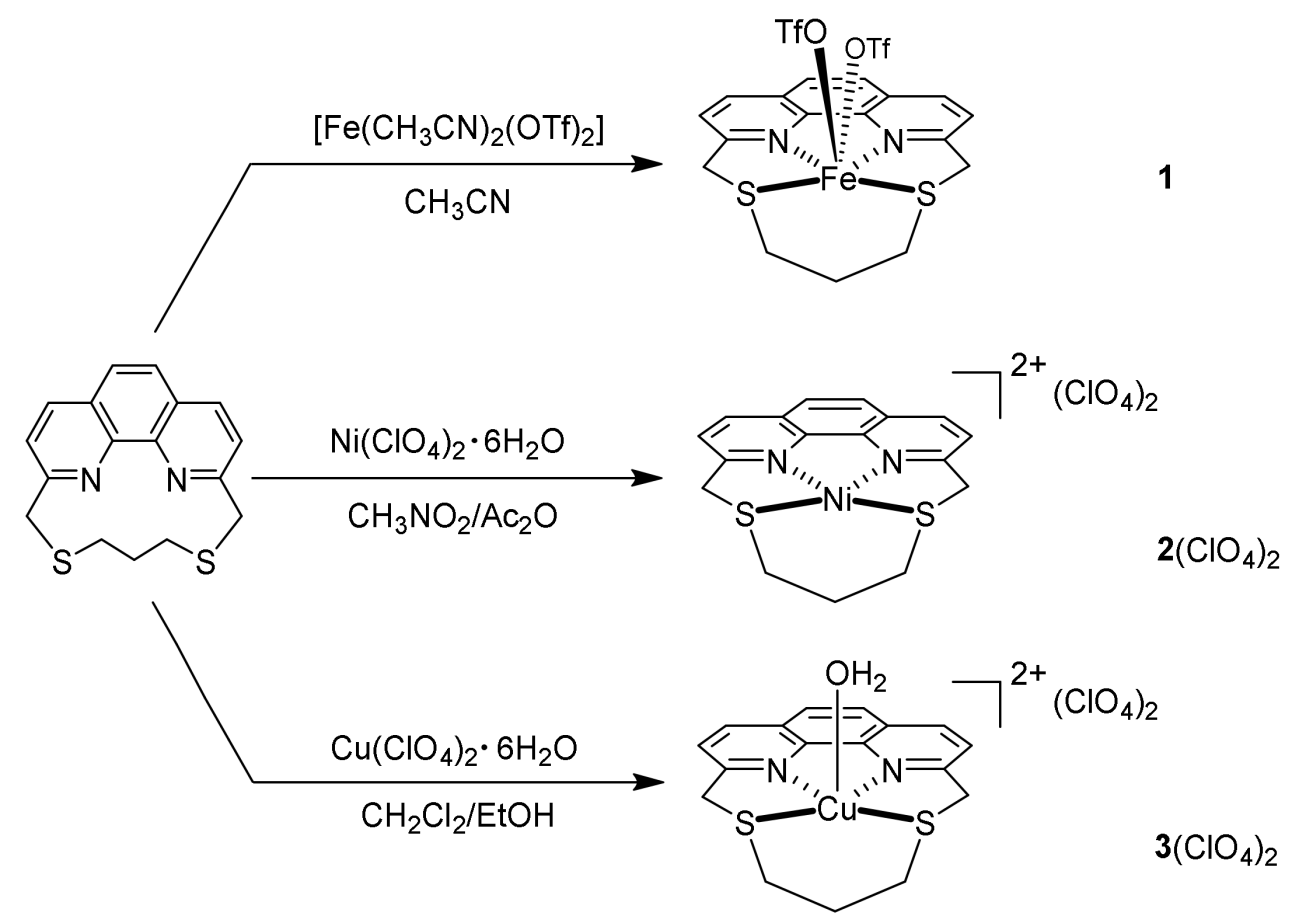
Synthesis of [13]ane(phenN_2_)S_2_-containing complexes **1**, **2**(ClO_4_)_2_ and **3**(ClO_4_)_2_.

**Figure 3:**
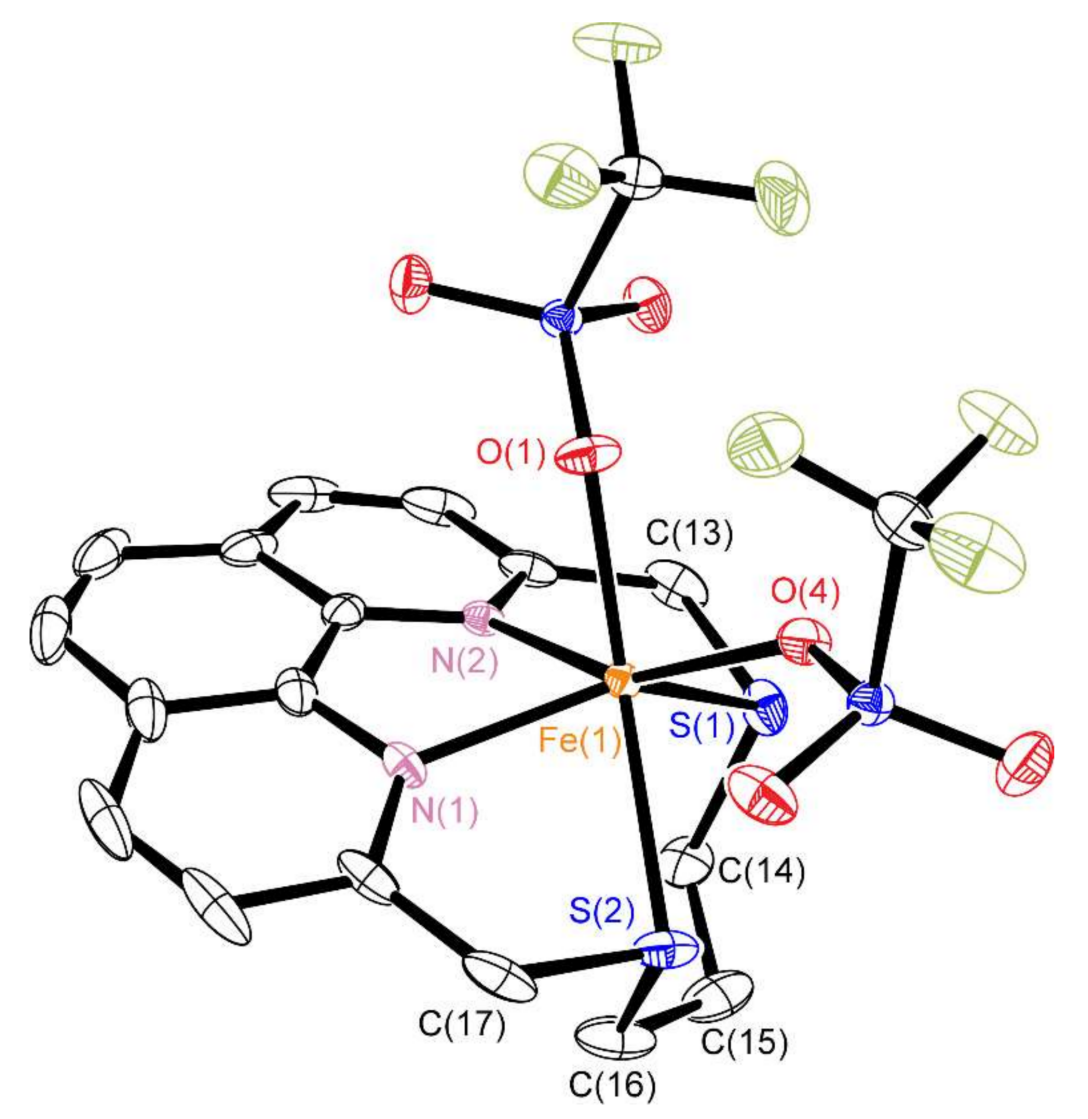
Perspective view of **1** as represented by 30% probability ellipsoids (hydrogen atoms on [13]ane(phenN_2_)S_2_ are omitted for clarity). Selected bond lengths (Å) and angles (°): Fe(1)-N(1), 2.156(2); Fe(1)-N(2), 2.209(2); Fe(1)-S(1), 2.486(1); Fe(1)-S(2), 2.531(1); Fe(1)-O(1), 2.134(2); Fe(1)-O(4), 2.126(2); N(1)-Fe(1)-N(2), 72.3(1); N(2)-Fe(1)-S(1), 74.5(1); S(1)-Fe(1)-S(2), 92.10(3); N(1)-Fe(1)-S(2), 75.4(1); O(1)-Fe(1)-N(1), 98.8(1); O(1)-Fe(1)-N(2), 81.9(1); O(4)-Fe(1)-S(1), 97.7(1); O(4)-Fe(1)-S(2), 82.6(1).

The coordination behavior of [13]ane(phenN_2_)S_2_ ligand was explored with various first-row transition metal ions including Fe(II), Ni(II) and Cu(II) (Scheme 3). Reacting metal precursors [Fe(CH_3_CN)_2_(OTf)_2_], Ni(ClO_4_)_2_·6H_2_O, and Cu(ClO_4_)_2_·6H_2_O with one equivalent of [13]ane(phenN_2_)S_2_ afforded a six-coordinated Fe complex [Fe([13]ane(phenN_2_)S_2_)(OTf)_2_] (**1**), four-coordinated Ni complex [Ni([13]ane(phenN_2_)S_2_)](ClO_4_)_2_ (**2**(ClO_4_)_2_), and five-coordinated Cu complex [Cu([13]ane(phenN_2_)S_2_)(OH_2_)](ClO_4_)_2_ (**3**(ClO_4_)_2_), respectively. All of them are isolable in good yields (70–80%). It is noteworthy that both **2**(ClO_4_)_2_ and **3**(ClO_4_)_2_ are very stable in CH_3_CN as their X-ray structures can be obtained upon recrystallization under ambient conditions. On the other hand, **1** exhibits a certain degree of instability towards air- and moisture-sensitive conditions. For example, the color of **1** changed from yellow to deep brown during recrystallization in CH_3_CN under ambient conditions, leading to an ill-defined product. Despite this observation, **1** cannot be regarded as very air-sensitive since its UV-visible absorption profile in non-degassed CH_3_CN remained unchanged for 16 h (Figure S1). The solubility of all metal complexes was also investigated in common organic solvents (CH_3_CN, acetone, CH_2_Cl_2_, THF and MeOH) and H_2_O. All of the three complexes were found to be soluble in CH_3_CN. In addition, **1** is soluble in acetone and CH_2_Cl_2_, whereas **2**(ClO_4_)_2_ and **3**(ClO_4_)_2_ are soluble in H_2_O. The solution magnetic moments for these complexes were determined via ^1^H-NMR spectroscopy using the Evans method [53,54]; the Fe(II) and Ni(II) complexes were determined to possess high-spin electronic states (*S* = 2 for Fe and *S* = 1 for Ni), whereas the electronic spin determined for the Cu(II) complex was the expected *S* = 1/2. The molecular structures for all these metal complexes were determined by X-ray crystallography. Their perspective views and selected structural parameters are depicted in Figure 3, Figures 5 and 6. It is evident that [13]ane(phenN_2_)S_2_ possesses structural flexibility to behave as a tetradentate ligand, and the energy required for the conformational change is expected to be small as complexes **1**, **2**(ClO_4_)_2_ and **3**(ClO_4_)_2_ can be prepared at room temperature.

**Figure 4:**
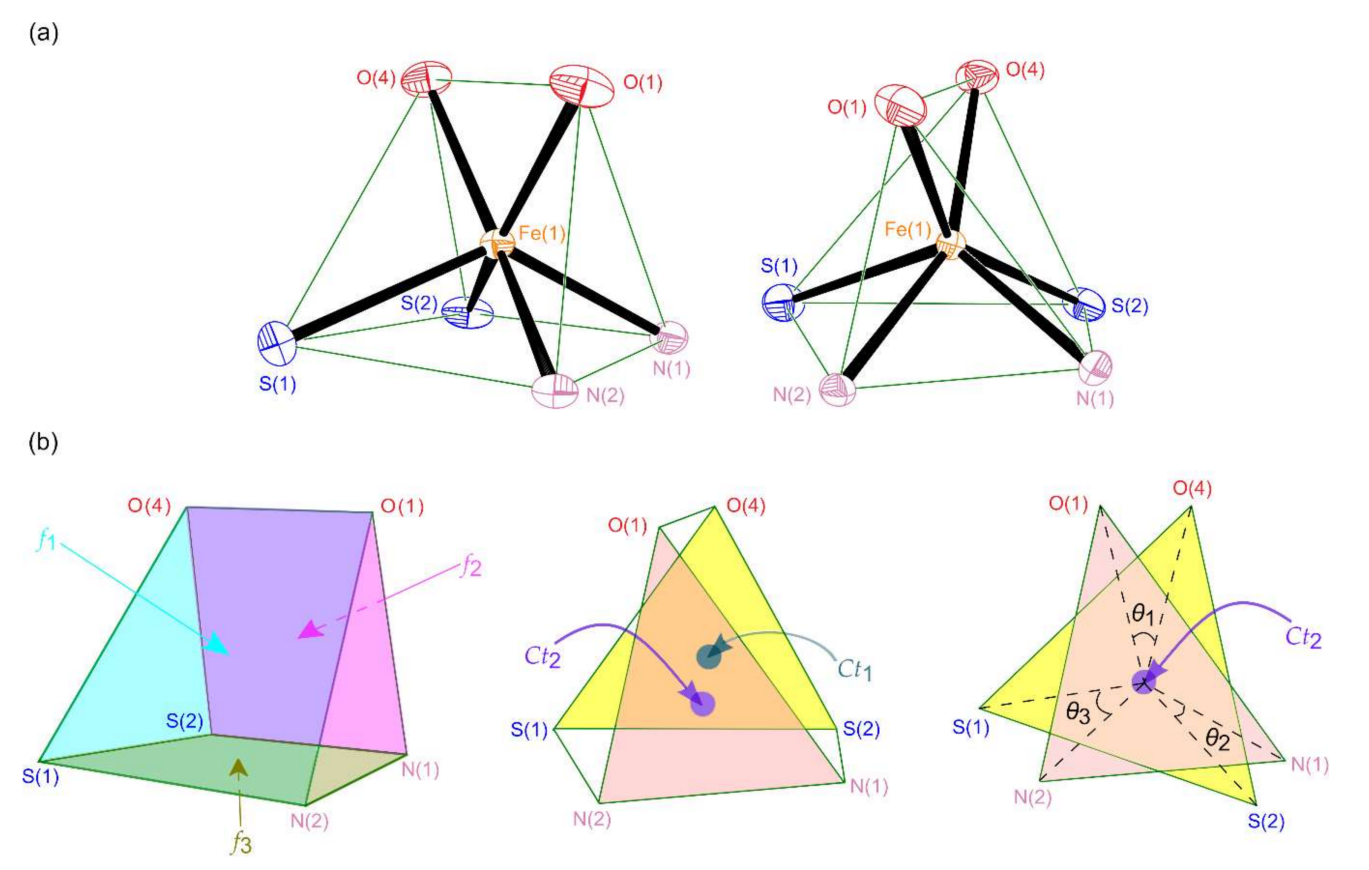
(**a**) Two different views of the coordination environment surrounding the Fe center of **1** and (**b**) illustrations of the three lateral faces (*f*_1_–*f*_3_), centroids (*Ct*_1_ and *Ct*_2_) and twist angles (*θ*_1_–*θ*_3_) of the two triangular basal planes on the twisted trigonal prism.

In high-spin (*S* = 2) Fe complex **1**, the [13]ane(phenN_2_)S_2_ acts as a tetradentate ligand, with two N and S atoms coordinating to the Fe center (Figure 3). Meanwhile, the Fe center is coordinated with two O-bound OTf. The coordination of OTf to the Fe center in solution was revealed by ^19^F-NMR study, with the observation of a single peak at −72.05 ppm indicating the coordinating nature of OTf [55,56,57]. The coordination geometry of **1** is best described as trigonal prismatic rather than octahedral. As depicted in Figure 4, the angles formed by the lateral faces *f*_1_ and *f*_2_ (61.5°), *f*_2_ and *f*_3_ (59.1°), *f*_3_ and *f*_1_ (60.5°) are all close to 60°, and the angle between the two triangular basal planes of the trigonal prism is small (7.9^o^), indicating that these basal planes are nearly parallel to each other. The distortion from a perfect trigonal prismatic geometry is characterized by the average twist angle of 14.9°. Meanwhile, the Fe-N (2.156(2)-2.209(2) Å) and Fe-S (2.486(1)-2.531(1) Å) bond distances are consistent with those of high-spin Fe(II) species bearing diimine moiety [58] and thioether linker [59]. The Fe-O bond distances (2.126(2)-2.134(2) Å) of **1** are typical of known high-spin Fe(II) complexes bearing coordinated OTf (2.025-2.211 Å) [60,61,62].

**Figure 5:**
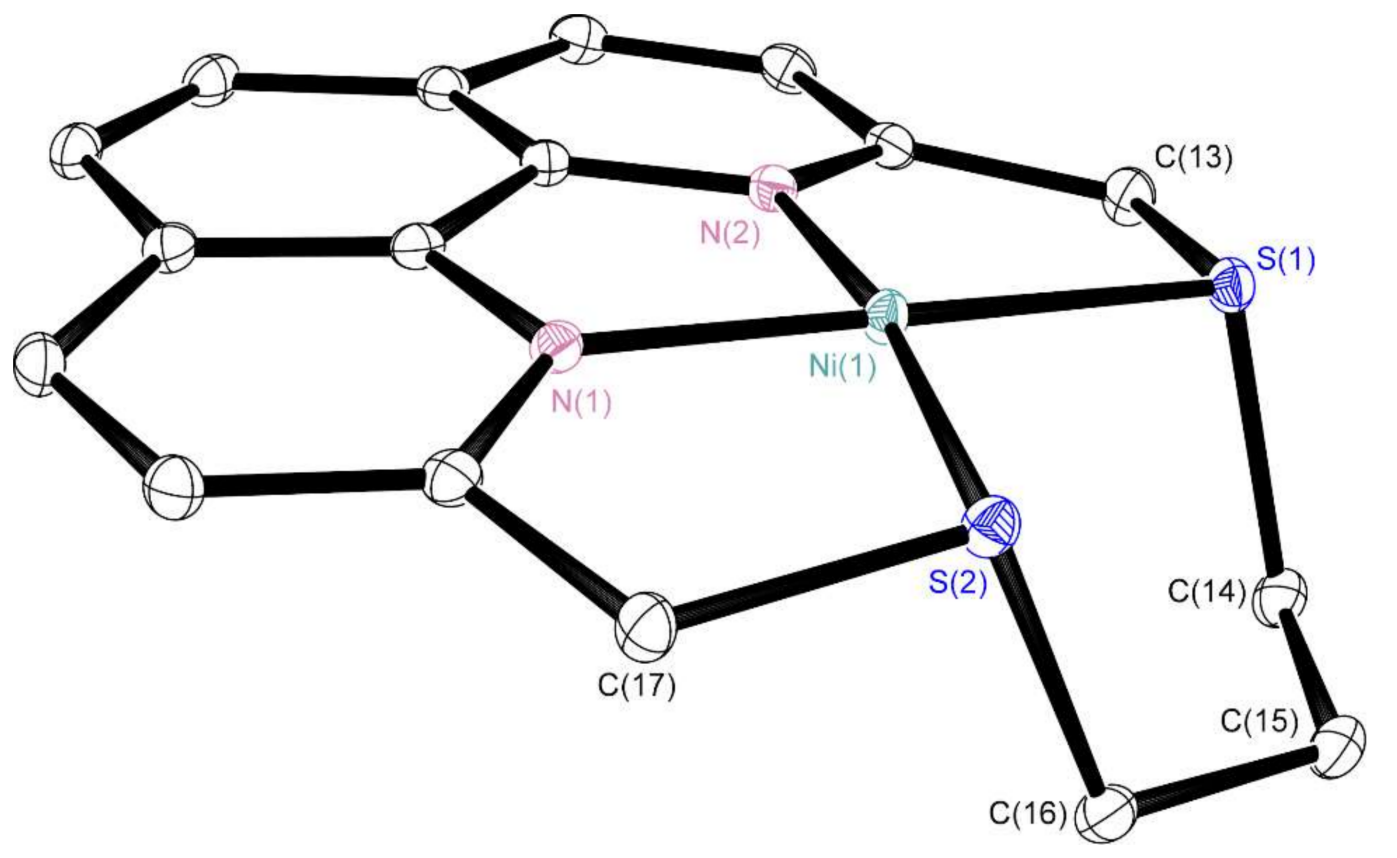
Perspective view of **2** as represented by 30% probability ellipsoids (hydrogen atoms on [13]ane(phenN_2_)S_2_ are omitted for clarity). Selected bond lengths (Å) and angles (°): Ni(1)-N(1), 1.849(2); Ni(1)-N(2), 1.855(2); Ni(1)-S(1), 2.155(1); Ni(1)-S(2), 2.156(1); N(1)-Ni(1)-N(2), 85.3(1); N(2)-Ni(1)-S(1), 87.5(1); S(1)-Ni(1)-S(2), 99.10(2); S(2)-Ni(1)-N(1), 87.8(1).

In high-spin (*S* = 1) **2**(ClO_4_)_2_, the Ni atom adopts a square planar geometry with the N and S atoms of [13]ane(phenN_2_)S_2_ occupying the equatorial position (Figure 5). The Ni atom only lies above the mean plane constructed by the four coordinating atoms of [13]ane(phenN_2_)S_2_ by 0.073 Å, and the geometry index *τ* [63,64] calculated for this complex is 0.11 (cf. *τ* ≈ 0 and *τ* ≈ 1 for square planar and tetrahedral geometry, respectively, in four-coordinated compounds). In comparison with [Ni([15]ane(phenN_2_)S_3_(CH_3_CN)]^2+^ where the Ni atom adopts a distorted octahedral geometry [46], the Ni-N and Ni-S bond distances in **2**(ClO_4_)_2_ (1.849(2)-1.855(2) Å for Ni-N, 2.155(1)-2.156(1) Å for Ni-S) are shorter than those in [Ni([15]ane(phenN_2_)S_3_(CH_3_CN)]^2+^ (2.013(2)–2.025(2) Å for Ni-N, 2.434(1)-2.444(1) Å for Ni-S). These observations are probably originated from the restricted flexibility of the shorter aliphatic linker in [13]ane(phenN_2_)S_2_.

**Figure 6:**
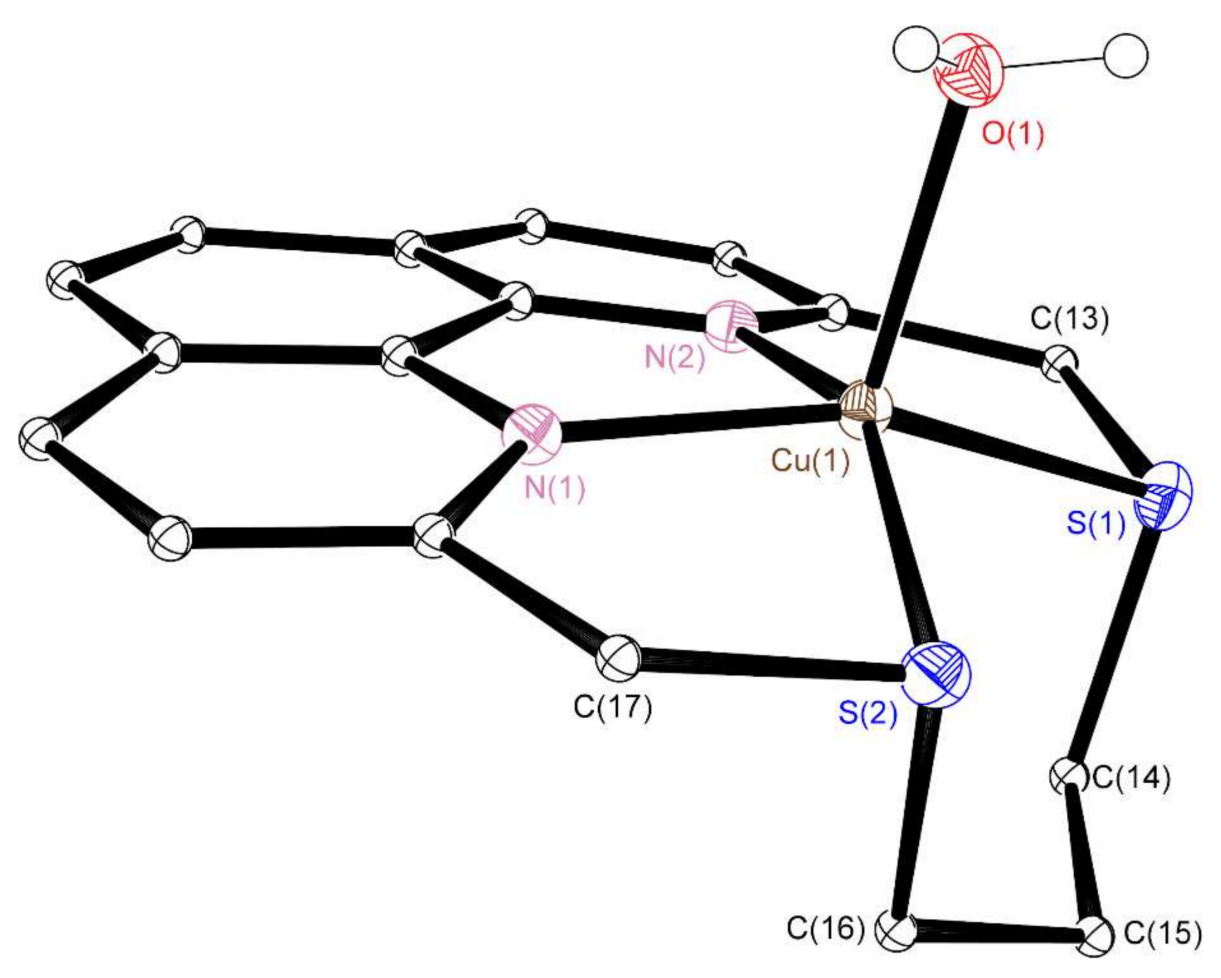
Perspective view of **3** as represented by 30% probability ellipsoids (hydrogen atoms on [13]ane(phenN_2_)S_2_ are omitted for clarity). Only one of the independent cations in [**3**(ClO_4_)_2_]_2_ is depicted. Selected bond lengths (Å) and angles (°) are listed in the order of Cu(1) moiety and then Cu(2) moiety: Cu(1)-N(1), 1.963(3), 1.950(3); Cu(1)−N(2), 1.959(3), 1.955(3); Cu(1)-S(1), 2.293(1), 2.293(1); Cu(1)-S(2), 2.292(1), 2.296(1); Cu(1)-O(1), 2.160(3), 2.165(3); N(1)-Cu(1)-N(2), 81.6(1), 82.1(2); N(2)-Cu(1)-S(1), 84.5(1), 84.4(1); S(1)-Cu(1)-S(2), 99.72(4), 100.51(4); S(2)-Cu(1)-N(1), 84.1(1), 84.2(1).

In **3**(ClO_4_)_2_, the Cu atom adopts a square pyramidal geometry (τ = 0.02; cf. τ ≈ 0 and τ ≈ 1 for square pyramidal and trigonal bipyramidal geometry, respectively, in five-coordinated compounds), with the N and S atoms of [13]ane(phenN_2_)S_2_ occupying the equatorial position, and the O atom of H_2_O in the axial position (Figure 6). The Cu atom is found to be located above the mean plane constructed by the four coordinating atoms of [13]ane(phenN_2_)S_2_ by 0.444 Å. The Cu-N bond distances of **3**(ClO_4_)_2_ (1.950(3)-1.963(3) Å) are comparable to that in [Cu([15]ane(phenN_2_)S_2_)(ClO_4_)]^+^ (1.956(5) Å) [47]. On the other hand, the Cu-S bond distances in **3**(ClO_4_)_2_ (2.292(1)-2.296(1) Å) are shorter than those in [Cu([15]ane(phenN_2_)S_2_)(ClO_4_)]^+^ (2.347(2) Å). This again reflects the restricted flexibility of the shorter aliphatic linker in [13]ane(phenN_2_)S_2_.

## 3. Materials and Methods

### 3.1. General Procedures

**Scheme 4:**
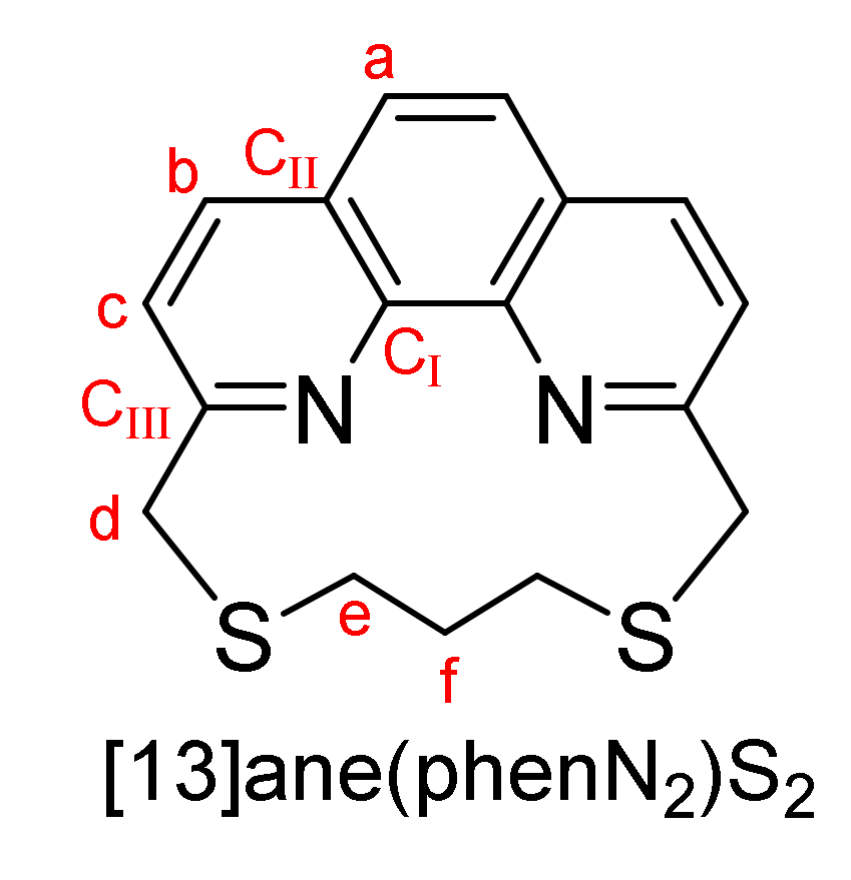
Labeling scheme for H and C atoms in [13]ane(phenN_2_)S_2_.

All reactions were performed under an argon atmosphere using standard Schlenk techniques unless otherwise stated. All reagents were used as received, and solvents for reactions were purified by a PureSolv MD5 solvent purification (Amesbury, MA, USA) system. The 2,9-dicarbaldehyde-1,10-phenanthroline [50,52], 2,9-bis(hydroxymethyl)-1,10-phenanthroline [51], 2,9-bis(chloromethyl)-1,10-phenanthroline [48,49] and Fe(CH_3_CN)_2_(OTf)_2_ [65], were prepared in accordance with the literature methods. ^1^H, ^13^C{^1^H}, ^1^H-^1^H COSY, ^1^H-^1^H-ROESY, ^1^H–^13^C-HSQC and ^1^H-^13^C-HMBC-NMR spectra were recorded on a Bruker 600 AVANCE III FT-NMR spectrometer (Billerica, MA, USA). Peak positions were calibrated with solvent residue peaks as internal standard. Labeling scheme for H and C atoms in the NMR assignments is shown in Scheme 4. Electrospray mass spectrometry was performed on a PE-SCIEX API 3200 triple quadrupole mass spectrometer (Tokyo, Japan). Elemental analyses were done on an Elementar Vario Micro Cube carbon-hydrogen-nitrogen elemental microanalyzer (Okehampton, UK). UV-visible spectra were recorded on a Shimadzu UV-1800 spectrophotometer (San Francisco, CA, USA). Neocuproine (98%), nickel(II) perchlorate hexahydrate (Ni(ClO_4_)_2_·6H_2_O) and copper(II) perchlorate hexahydrate (Cu(ClO_4_)_2_·6H_2_O) were purchased from J&K Scientific Ltd (Hong Kong). Iron(II) chloride (FeCl_2_, anhydrous, 98%) and trimethylsilyl trifluoromethanesulphonate ((CH_3_)_3_SiOTf, 98%) were purchased from Fluorochem Ltd. (Derbyshire, UK)

### 3.2. Solution Magnetic Susceptibility Measurements

The solution state molar magnetic susceptibility ($\chi_{m}$) of **1**, **2**(ClO_4_)_2_ and **3**(ClO_4_)_2_ was measured according to the Evans method [53,54]. This involves the placement of a coaxial insert containing sample solution (0.010 M) into a standard 5 mm NMR tube containing only solvent. CD_3_CN containing one drop of benzene as internal reference was used as the solvent throughout. Based on the chemical shift difference of the benzene internal reference between inner and outer tubes, values of molar magnetic susceptibility ($\chi_{m}$) and magnetic moment ($\mu_{eff}$) were calculated using the equations shown below [66,67]:

(1) $$\chi_{m}\;=\;\left(3\cdot \Delta f\right)/\left(1000\cdot f\cdot c\right)$$

(2) $$\mu_{eff}\;=\;798\sqrt{T\cdot \chi_{m}}\;$$

where $\chi_{m}$ = molar magnetic susceptibility of the sample (m^3^ mol^−1^); △f = difference of the chemical shift (Hz) between the internal references of inner and outer tubes; f = operating frequency of the NMR spectrometer (Hz); c = concentration of the metal complex (mol dm^−3^); T = probe temperature of the measurement (K).

### 3.3. Geometry Index

The geometry index (τ) is a parameter ranging from 0 to 1 to describe the geometry of the coordination center in four- and five-coordinated compounds.

For four-coordinated compounds, the τ is used to distinguish whether the coordination geometry of the metal center is square planar (τ ≈ 0) or tetrahedral (τ ≈ 1) [64]. The formula is shown below:

(3) $$\tau =\left(\beta -\alpha \right)/\left(360{}^{\circ}-\theta \right)+\left(180{}^{\circ}-\beta \right)/\left(180{}^{\circ}-\theta \right)\approx -0.00399\alpha -0.01019\beta +2.55$$

where α and β are the two largest valence angles of the coordination center (β > α) and θ = cos^−1^ (−1/3) ≈ 109.5° is a tetrahedral angle.

For **2**(ClO_4_)_2_, α(N(2)-Ni(1)-S(2)) = 171.9° and β(N(1)-Ni(1)-S(1)) = 172.0°.

For five-coordinated compounds, the τ is used to distinguish whether the coordination geometry of the metal center is square pyramidal (τ ≈ 0) or trigonal bipyramidal (τ ≈ 1) [63]. The formula is shown below:

(4) $$\tau =\left(\beta -\alpha \right)/60{}^{\circ}\approx -0.01667\alpha +0.01667\beta$$

where α and β are the two largest valence angles of the coordination center (β > α).

For **3**(ClO_4_)_2_, α(N(1)-Cu(1)-S(1)) = 154.0° and β(N(2)-Cu(1)-S(2)) = 155.0°.

### 3.4. Synthesis of ([13]ane(phenN_2_)S_2_) and [13]ane(phenN_2_)S_2_-containing Fe(II), Ni(II), Cu(II) complexes

*Synthesis of 2,9-dicarbaldehyde-1,10-phenanthroline*. The ligand was synthesized from a modified literature procedure [52]: To a solution of selenium oxide (2.26 g, 20.41 mmol) in deionized water (5 mL) and 1,4-dioxane (70 mL), 2,9-dimethyl-1,10-phenanthroline (2.00 g, 9.60 mmol) in 1,4-dioxane (20 mL) was added in a dropwise manner. The reaction mixture was refluxed for 0.5 h, during which the color of the reaction mixture changed from yellow to reddish brown with black deposits. Upon cooling to room temperature, the brown solids were collected by suction filtration and washed with Et_2_O (10 mL × 3). These solids were further purified by recrystallization with warm CHCl_3_ and allowed to cool and stand overnight. Yellowish orange solids were collected by suction filtration and washed with Et_2_O (10 mL × 3) to give 2,9-dicarbaldehyde-1,10-phenanthroline. ^1^H-NMR signals are consistent with that of the literature reported [50]. Yield: 1.95 g, 86%.

*Synthesis of 2,9-bis(hydroxymethyl)-1,10-phenanthroline*. The ligand was synthesized from a modified literature procedure [51]: To a solution of 2,9-dicarbaldehyde-1,10-phenanthroline (1.60 g, 6.77 mmol) in absolute EtOH (120 mL), anhydrous NaBH_4_ (1.02 g, 27.09 mmol) was added. The reaction mixture was refluxed under argon for 1.5 h, during which the color of the reaction mixture changed from yellow to brown. Upon cooling to room temperature, the reaction mixture was removed by reduced pressure. The aqueous layer was extracted with EtOAc (50 mL × 2). The organic phases were combined, washed with brine (50 mL × 2), dried over anhydrous MgSO_4_, and concentrated to give 2,9-bis(hydroxymethyl)-1,10-phenanthroline as pale pink solids. ^1^H-NMR signals are consistent with that of literature reported. Yield: 1.20 g, 74%.

*Synthesis of 2,9-bis(chloromethyl)-1,10-phenanthroline.* The ligand was synthesized from a modified literature procedure [49]: To a SOCl_2_ solution (16 mL) at 0 °C, 2,9-bis(hydroxymethyl)-1,10-phenanthroline (1.60 g, 6.66 mmol) was added. The reaction mixture was stirred for 2 h at 0 °C, during which the color of the reaction mixture changed from colorless to yellow. Upon adding *n*-hexane (30 mL) to the reaction mixture, the deep yellow precipitates were collected, dissolved in CHCl_3_ and extracted with an aqueous layer containing 5% (*w*/*v*) Na_2_CO_3_ and 5% (*w*/*v*) NaHCO_3_ for neutralization. The organic phases were combined, washed with brine (50 mL × 2), dried over anhydrous MgSO_4_ and concentrated to give 2,9-bis(chloromethyl)-1,10-phenanthroline as yellow solids. ^1^H-NMR signals are consistent with that of literature reported [48]. Yield: 1.52 g, 82%.

*Synthesis of 2,6-dithia[7](2,9)-1,10-phenanthrolinophane ([13]ane(phenN_2_)S_2_).* To a well-stirred suspension of dry Cs_2_CO_3_ (3.34 g, 10.24 mmol) in anhydrous DMF (224 mL) at 55 °C, a mixture of 1,3-propanedithiol (0.51 mL, 5.12 mmol) and 2,9-bis(chloromethyl)-1,10-phenanthroline (1.43 g, 5.12 mmol) in anhydrous DMF (112 mL) was added under argon for 42 h at a rate of 2.67 mL/min, during which the color of the reaction mixture changed from colorless to pale yellow suspension. Upon cooling to room temperature over a period of 24 h, the reaction mixture was removed by vacuum distillation. After dissolving the residue in CH_2_Cl_2_, the pale orange suspension was filtered to remove Cs_2_CO_3_. The filtrate was concentrated to give a red oil. The [13]ane(phenN_2_)S_2_ was obtained as white solids after silica gel column chromatography (silica gel, gradual elution with CH_2_Cl_2_/EtOAc (8:2, *v*/*v*); R_f_ value = 0.70 (silica gel TLC; CH_2_Cl_2_/EtOAc (8:2, *v*/*v*))). Recrystallization by slow diffusion of Et_2_O solution into a CH_2_Cl_2_ solution of the white solids gave analytically pure [13]ane(phenN_2_)S_2_ as white crystals. Yield: 0.45 g, 30%. Anal. Calcd. for C_17_H_16_N_2_S_2_: C, 65.35; H, 5.16; N, 8.97. Found: C, 65.38; H, 5.18; N, 8.99. ESI-MS found (calcd.): *m*/*z* 313.30 (313.46) [C_17_H_16_N_2_S_2_ + H]^+^. ^1^H-NMR (600 MHz, CDCl_3_): δ 2.83–2.98 (m, 2H, H_f_), 3.11–3.22 (m, 4H, H_e_), 4.12–4.29 (m, 4H, H_d_), 7.43 (d, *J* = 6.0 Hz, 2H, H_b_), 7.73 (s, 2H, H_a_), 8.14 (d, *J* = 6.0 Hz, 2H, H_c_). ^13^C-NMR (150 MHz, CDCl_3_): δ 34.57 (C_f_), 36.72 (C_e_), 41.81 (C_d_), 121.71 (C_b_), 125.73 (C_a_), 127.37 (C_II_), 136.35 (C_c_), 146.07 (C_I_), 160.79 (C_III_).

*Synthesis of [Fe(CH_3_CN)_2_(OTf)_2_].* The metal precursor was synthesized from a modified literature procedure [65]: To a solution of FeCl_2_ (0.50 g, 3.94 mmol) in anhydrous CH_3_CN (20 mL), (CH_3_)_3_SiOTf (4 mL in 10 mL anhydrous CH_3_CN, 22.10 mmol) was added in a dropwise manner. The reaction mixture was stirred under argon for 16 h, during which the color of the reaction mixture changed from pale yellow to colorless. The colorless solution was concentrated to about 5 mL by reduced pressure, and then added to Et_2_O (150 mL) to give white precipitates. The solids were collected by suction filtration, washed with Et_2_O (10 mL × 3) and dried by reduced pressure to give [Fe(CH_3_CN)_2_(OTf)_2_]. Yield: 1.20 g, 70%.

*Synthesis of **1**.* A mixture of [Fe(CH_3_CN)_2_(OTf)_2_] (0.40 g, 0.91 mmol) and [13]ane(phenN_2_)S_2_ (0.28 g, 0.91 mmol) was stirred in CH_3_CN (30 mL) under argon for 16 h, during which the color of the reaction mixture changed from red to deep red with pale yellow precipitates. Upon filtration for removal of these precipitates, the supernatant was collected and concentrated to give deep red precipitates. The solids were collected by suction filtration and washed with Et_2_O (10 mL × 3). Recrystallization by slow diffusion of Et_2_O solution into a CH_3_CN solution of the deep red solids under argon gave analytically pure **1** as orange crystals. Yield: 0.43 g, 71%. Anal. Calcd. for C_19_H_16_N_2_S_4_FeF_6_O_6_: C, 34.24; H, 2.42; N, 4.20. Found: C, 34.28; H, 2.44; N, 4.21. ^19^F-NMR (400 MHz, CD_3_CN): δ −72.05. ESI-MS found (calcd.): *m*/*z* 517.30 (517.38) [C_18_H_16_N_2_S_3_FeF_3_O_3_]^+^. Evans NMR at 298 K: *μ_eff_* = 4.98 *μ*_B_.

*Synthesis of **2**(ClO_4_)_2_.* To a solution of Ni(ClO_4_)_2_·6H_2_O (0.04 g, 0.10 mmol) in nitromethane (15 mL) and acetic anhydride (0.62 mL), [13]ane(phenN_2_)S_2_ (0.03 g, 0.10 mmol) was added. The reaction mixture was stirred under argon for 2 h, during which the color of the reaction mixture changed from pale green to yellow. The reaction mixture was concentrated to about 5 mL by reduced pressure and then added to Et_2_O (150 mL) to give orange precipitates. The solids were collected by suction filtration, washed with deionized water (5 mL × 2), cold EtOH (5 mL × 2), and finally Et_2_O (10 mL × 3). Recrystallization by slow diffusion of Et_2_O into a CH_3_CN solution of the orange precipitates gave analytically pure **2**(ClO_4_)_2_ as brown crystals. Yield: 0.05 g, 80%. Anal. Calcd. for C_17_H_16_N_2_S_2_NiCl_2_O_8_: C, 35.82; H, 2.83; N, 4.91. Found: C, 35.85; H, 2.86; N, 4.93. ESI-MS found (calcd.): *m*/*z* 470.30 (470.60) [C_17_H_16_N_2_S_2_NiClO_4_]^+^. Evans NMR at 298 K: *μ_eff_* = 2.77 *μ*_B_.

*Synthesis of **3**(ClO_4_)_2_.* To a solution of a mixture of [13]ane(phenN_2_)S_2_ (0.03 g, 0.10 mmol) in EtOH/CH_2_Cl_2_ (10 mL, 2:1 (*v*/*v*)), Cu(ClO_4_)_2_·6H_2_O (0.89 g, 2.40 mmol) in EtOH (5 mL) was added. The reaction mixture was stirred under argon for 4 h, during which the color of the reaction mixture changed from pale blue to pale purple with bluish green precipitates. The solids were collected by suction filtration, washed with cold EtOH (5 mL × 2), and finally Et_2_O (10 mL × 3). Recrystallization by slow diffusion of Et_2_O into a CH_3_CN solution of the bluish green precipitates gave analytically pure **3**(ClO_4_)_2_ as purple crystals. Yield: 1.00 g, 70%. Anal. Calcd. for C_17_H_18_N_2_S_2_CuCl_2_O_9_: C, 34.44; H, 3.06; N, 4.72. Found: C, 34.48; H, 3.08; N, 4.76. ESI-MS found (calcd.): *m*/*z* 475.30 (475.45) [C_17_H_16_N_2_S_2_CuClO_4_]^+^. Evans NMR at 298 K: *μ_eff_* = 1.69 *μ*_B_.

### 3.5. X-ray Crstayllographic Data

CCDC 1994366–1994369 contain the supplementary crystallographic data for this paper. These data can be obtained free of charge via http://www.ccdc.cam.ac.uk/conts/retrieving.html (or from the CCDC, 12 Union Road, Cambridge CB2 1EZ, UK; Fax: +44 1223 336033; E-mail: deposit@ccdc.cam.ac.uk).

Crystal Data for [13]ane(phenN_2_)S_2_ (C_17_H_16_N_2_S_2_; *M* = 312.44 g/mol): triclinic, space group P-1 (no. 2), *a* = 8.7121(4) Å, *b* = 10.2500(6) Å, *c* = 10.4864(5) Å, *α* = 62.718(2)°, *β* = 68.073(2)°, *γ* = 70.672(2)°, *V* = 757.41(7) Å^3^, *Z* = 2, *T* = 212.99 K, μ(MoKα) = 0.346 mm^−1^, *Dcalc* = 1.370 g/cm^3^, 9505 reflections measured (4.558° ≤ 2Θ ≤ 52.754°), 3087 unique (*R*_int_ = 0.0502, R_sigma_ = 0.0514) which were used in all calculations. The final *R*_1_ was 0.0393 (I > 2σ(I)) and *wR*_2_ was 0.0973 (all data). (CCDC 1994366)

Crystal Data for **1** (C_19_H_16_F_6_O_6_S_4_N_2_Fe; *M* = 666.43 g/mol): monoclinic, space group C2/c (no. 15), *a* = 28.3742(8) Å, *b* = 9.9821(3) Å, *c* = 18.6161(5) Å, *β* = 112.3340(10)°, *V* = 4877.2(2) Å^3^, *Z* = 8, *T* = 172.98 K, μ(MoKα) = 1.048 mm^−1^, *Dcalc* = 1.815 g/cm^3^, 29,372 reflections measured (4.366° ≤ 2Θ ≤ 52.764°), 4989 unique (*R*_int_ = 0.0523, R_sigma_ = 0.0336) which were used in all calculations. The final *R*_1_ was 0.0367 (I > 2σ(I)) and *wR*_2_ was 0.0898 (all data). (CCDC 1994367)

Crystal Data for **2**(ClO_4_)_2_ (C_17_H_16_N_2_O_8_S_2_Cl_2_Ni; *M* = 570.05 g/mol): triclinic, space group P-1 (no. 2), *a* = 8.8218(7) Å, *b* = 10.0103(8) Å, *c* = 12.7982(9) Å, *α* = 108.789(7)°, *β* = 106.996(7)°, *γ* = 92.621(6)°, *V* = 1010.95(13) Å^3^, *Z* = 2, *T* = 293(2) K, μ(CuKα) = 6.231 mm^−1^, *Dcalc* = 1.873 g/cm^3^, 6468 reflections measured (7.72° ≤ 2Θ ≤ 134.98°), 3576 unique (*R*_int_ = 0.0202, R_sigma_ = 0.0256) which were used in all calculations. The final *R*_1_ was 0.0295 (I > 2σ(I)) and *wR*_2_ was 0.0810 (all data). (CCDC 1994368)

Crystal Data for **3**(ClO_4_)_2_ (C_17_H_18_N_2_O_9_S_2_Cl_2_Cu; *M* = 592.89 g/mol): monoclinic, space group P2_1_/c (no. 14), *a* = 26.9718(6) Å, *b* = 11.0279(3) Å, *c* = 14.7341(3) Å, *β* = 99.779(2)°, *V* = 4318.87(18) Å^3^, *Z* = 8, *T* = 293(2) K, μ(MoKα) = 1.508 mm^−1^, *Dcalc* = 1.824 g/cm^3^, 20,460 reflections measured (6.62° ≤ 2Θ ≤ 53°), 8925 unique (*R*_int_ = 0.0213, R_sigma_ = 0.0291) which were used in all calculations. The final *R*_1_ was 0.0487 (I > 2σ(I)) and *wR*_2_ was 0.1291 (all data). (CCDC 1994369)

### 3.6. Computational Methodology

The structures and relative energies of Conformers **I**−**III** were calculated by density functional theory (DFT) calculations using the ORCA software package (version 4.1.1, Max-Planck-Institut für Chemische Energiekonversion, Mülheim an der Ruhr, North Rhine-Westphalia, Germany) [68]. All the calculated structures were optimized in the gas phase at the BP86/def2-SVP level with the Grimme’s DFTD3 method to account for the dispersion effects, and the Resolution of Identity (RI) approximation to speed up the calculations. Tight self-consistent field (SCF) convergence criteria were used throughout. The optimized conformers were confirmed to be local minima via frequency calculations (no negative frequency).

## 4. Conclusions

A new tetradentate mixed aza-thioether macrocyclic ligand 2,6-dithia[7](2,9)-1,10-phenanthrolinophane ([13]ane(phenN_2_)S_2_) was successfully prepared. Three low-valent first-row transition metal complexes supported by [13]ane(phenN_2_)S_2_, namely [Fe([13]ane(phenN_2_)S_2_)(OTf)_2_], [Ni([13]ane(phenN_2_)S_2_)](ClO_4_)_2_ and [Cu([13]ane(phenN_2_)S_2_)(OH_2_)](ClO_4_)_2_, were synthesized from reactions between corresponding transition metal precursors and [13]ane(phenN_2_)S_2_ under mild reaction conditions. The X-ray structures of the metal complexes revealed that [13]ane(phenN_2_)S_2_ acts as a tetradentate ligand by coordinating to the metal centers via donor atoms N and S. The success of preparing these macrocyclic complexes undeniably opens new opportunities for further reactivity studies and possible biological applications.

## Figures and Tables

**Table 1:** Selected bond lengths (Å) and angles (°) for Conformers **I**–**III**.

Conformers	I	II	III
N(1)-C(1)	1.332	1.332	1.331
N(1)-C(5)	1.349	1.348	1.348
N(2)-C(6)	1.349	1.353	1.343
N(2)-C(10)	1.332	1.327	1.330
C(1)-C(17)	1.511	1.506	1.512
C(10)-C(13)	1.511	1.522	1.511
S(1)-C(13)	1.844	1.825	1.850
S(1)-C(14)	1.845	1.850	1.846
S(2)-C(16)	1.846	1.838	1.851
S(2)-C(17)	1.846	1.851	1.856
C(1)-C(17)-S(2)	114.5	112.9	111.6
C(10)-C(13)-S(1)	114.4	116.9	111.9
C(13)-S(1)-C(14)	102.7	101.7	101.8
C(16)-S(2)-C(17)	102.6	103.6	105.1

## Supplementary Materials

The following are available online, Table S1–S3: Cartesian coordinates of calculated structures of Conformers **I**–**III**. Figure S1: UV-visible absorption spectra of **1**, **2**(ClO_4_)_2_ and **3**(ClO_4_)_2_ in CH_3_CN at 298 K, Figure S2: ^1^H-NMR spectrum of [13]ane(phenN_2_)S_2_, Figure S3: ^13^C{^1^H}-NMR spectrum of [13]ane(phenN_2_)S_2_, Figure S4: ^1^H-^1^H COSY of [13]ane(phenN_2_)S_2_, Figure S5: ^1^H-^1^H ROESY of [13]ane(phenN_2_)S_2_, Figure S6: ^1^H-^13^C HSQC of [13]ane(phenN_2_)S_2_, Figure S7: ^1^H-^13^C HMBC of [13]ane(phenN_2_)S_2_.
