# Supplementary material for: A New Tetradentate Mixed Aza-Thioether Macrocycle and Its Complexation Behavior towards Fe(II), Ni(II) and Cu(II) Ions
Source: Molecules. 2020 Apr 27;25(9):2030. doi: 10.3390/molecules25092030 (PMC7248963; doi:10.3390/molecules25092030)
Supplement: Supplementary file 1 [file molecules-25-02030-s001.pdf]

Supporting Information

A New Tetradentate Mixed Aza-Thioether Macrocycle And Its Complexation Behavior Towards Fe(II), Ni(II) and Cu(II) Ions

Sze-Wing Ng [1,2,†], Siu-Chung Chan [1,†], Chi-Fung Yeung [1,2], Shek-Man Yiu [1] and Chun-Yuen Wong [1,2*]

[1] Department of Chemistry, City University of Hong Kong, Tat Chee Avenue, Kowloon, Hong Kong SAR

[2] State Key Laboratory of Terahertz and Millimeter Waves, City University of Hong Kong, Tat Chee Avenue, Kowloon, Hong Kong SAR

† These authors contributed equally to this work.

E-mail: acywong@cityu.edu.hk

Table of Contents

	Page
Computational Details	2
UV-visible Absorption Spectra of **1**, **2**(ClO_4)$_2$ and **3**(ClO_4)$_2$	8
NMR Spectra for [13]ane(phenN_2)S_2	9

Table S1. Cartesian coordinates of Conformer **I** at the BP86 optimized geometry

	Coordinates in Å		
	X	Y	Z
S	9.22864558337117	1.65330083756436	5.69355668047001
S	5.70721976189211	4.86479477938505	8.86560247180710
N	7.24036970622651	3.71622091210090	4.10354758096137
N	5.55912380219693	5.25117805118648	5.61978105106928
C	8.13460615762977	2.97846647370244	3.44704852198588
C	8.78275134803536	3.45010801709112	2.26981410556879
H	9.51318112828641	2.81301936434864	1.74830273149622
C	8.47653807110207	4.72006118580843	1.79330368487409
H	8.95424991007210	5.11062518899501	0.88123787021569
C	7.55259005920401	5.53376451674661	2.50068481639730
C	7.21797435239934	6.87613224130483	2.10524633797762
H	7.66720527074329	7.28124617542322	1.18529914447055
C	6.37302340362020	7.64866435098277	2.86849238359837
H	6.13750332658466	8.68186748023496	2.56957519674071
C	5.79793242454489	7.13675899910914	4.08406844791786
C	4.98085979533483	7.91368712432276	4.94734145277712
H	4.74669840264038	8.95579619350152	4.67946599418675
C	4.50396187923148	7.35821692769769	6.12959672569498
H	3.88945515932704	7.95136242415829	6.82390093878579
C	4.82164928244251	6.00379262574868	6.43475904403786
C	6.06471146367693	5.78608376313241	4.48917855624426
C	6.96238625605485	4.96631224107110	3.67935352142666
C	8.43546738980996	1.61644158862977	4.02758365334929
H	9.13808675948829	1.05396543857349	3.38136396801804
H	7.49955130472284	1.02624849120777	4.11231766665391
C	7.77846865751661	1.79825230272449	6.82660228378151
H	6.91260149200682	1.33423574747340	6.30841593183273
H	8.02726004806654	1.14066483904581	7.68766513865121
C	7.43878915704232	3.21901843958776	7.28836425926210
H	7.20373680198694	3.83263374789470	6.39976608579006

H	8.31193766838819	3.67055158714928	7.80472951741257
C	6.22200509150861	3.21519377535186	8.21952861046442
H	6.40984702653414	2.60269542540432	9.12824162576471
H	5.33846868683449	2.77362853821864	7.71226316609480
C	4.35141384735477	5.34718566327597	7.71184883487324
H	3.76123391363332	4.43687664414924	7.47653009178526
H	3.70229561048930	6.03044789769709	8.29472190756187

**Table S2.** Cartesian coordinates of Conformer **II** at the BP86 optimized geometry			
	Coordinates in Å		
	X	Y	Z
S	8.55558942315099	1.55081984421324	6.20615730154080
N	7.63388343306937	3.86362931397712	4.47533867459896
N	5.96954767240881	5.44263168726128	5.94525318824998
C	8.46488773924101	3.08285728982095	3.79636549027136
C	8.92497078523868	3.42718624988763	2.48892171461677
H	9.62526781451737	2.76441106705312	1.95643581996237
C	8.46772366388056	4.59799365121092	1.89740088914221
H	8.79863631348383	4.88505473258366	0.88706147711961
C	7.55137180105908	5.43090072919602	2.59761492852644
C	6.97866585262820	6.62978580854359	2.04416451853753
H	7.27856318892573	6.93673429755570	1.03016362089313
C	6.05541071273074	7.36836487587859	2.75139661966479
H	5.60705556379367	8.27130823028593	2.30828097271185
C	5.65647233835095	6.97964686446786	4.07766159451756
C	4.68303523547768	7.67380983867039	4.84926348040452
H	4.18844488139546	8.56067633692867	4.42273769562634
C	4.36147944385868	7.22767746133704	6.12650313050997
H	3.59786835858398	7.74158933983236	6.72978634322281
C	5.05959809445081	6.10415961294288	6.65856697334360
C	6.24906491010154	5.82381163260976	4.68303903132202
C	7.18221502495533	5.00988413020130	3.91548730341665
C	8.87306155852445	1.75607237071663	4.42039751423504
H	9.96609980986969	1.60037096010932	4.30166071594322
H	8.38855278218344	0.92881437905800	3.85414843585358
C	6.70644166019205	1.55290695236196	6.25947750781303
C	4.82250041210004	5.55653281782687	8.04132061963060
H	4.25964216243303	6.26384108437521	8.68157299886922
S	3.77797325143588	4.02858230910629	8.04196090019001
H	5.79613073052842	5.33841288046940	8.52355418866670
C	4.67820819474654	2.89140007336458	6.91282515608351

H	4.60934762867789	3.29049888250790	5.87920488286293
H	4.05064357242467	1.97432618376852	6.95531045445522
C	6.14508134387276	2.59424586405640	7.23843604955813
H	6.36262324561700	1.79095770462712	5.23474520533570
H	6.36202207053643	0.52652565440217	6.50790723228710
H	6.72076005086325	3.52971275460865	7.10022182499651
H	6.25035420469199	2.25384385418297	8.29013204502022

Table S3. Cartesian coordinates of Conformer **III** at the BP86 optimized geometry

	Coordinates in Å		
	X	Y	Z
C	0.74384438196776	7.15326358370636	12.59882164629804
C	-0.29309053061961	6.19169850770784	12.77797574925819
H	-0.04861472687241	5.12397541465267	12.88818344118351
C	-1.61744835930712	6.62595739277213	12.80775958173085
H	-2.44098606439883	5.90303487940361	12.92261694011229
C	-1.91106740843718	8.01655907653523	12.73712792156820
C	-0.78865872091860	8.89191276432984	12.59287621942580
C	-0.97532368665204	10.33260006642702	12.69533808531487
C	-2.29792422904908	10.85485554332004	12.86881076999846
C	-2.40225249431182	12.25987496169305	13.05027292902504
H	-3.39470248772726	12.72049905355943	13.17884439375398
C	-1.24713647697736	13.03667551614738	13.10527389294132
H	-1.30761620323813	14.12171562543747	13.28147715028266
C	0.02100107209503	12.40550124163619	12.95513771832216
C	-3.23110864529701	8.58224182739976	12.85067311184662
H	-4.09476221877201	7.90215664713359	12.92181197477497
C	-3.41661951216045	9.94711465612126	12.90299164477602
H	-4.42970381425236	10.36567138886447	13.01139046892227
C	1.32336637745337	13.15653877891311	13.11328411652184
H	1.16090434739743	14.25207178843714	13.14330030330569
H	1.99799447329775	12.92132488637500	12.26666603179933
C	3.74268688113193	11.81468277806963	14.10955223803615
H	3.85970360354868	11.99924736165735	13.02152870605392
H	4.57007262695636	12.35034898331067	14.61728718497379
C	3.82280977488558	10.31250889966674	14.43865167584954
H	4.89764195815349	10.02759155809194	14.49300930163075
H	3.40684822004384	10.14292119459949	15.45566070330769
C	3.09604068181243	9.43140938423498	13.42348959958016
H	3.59977059405149	9.48199510829225	12.43274205039466
H	2.04822899177334	9.76351269428919	13.27641477514230

C	2.21901579239733	6.82551020777459	12.60654561616979
H	2.68417150139896	7.13462008759856	11.64645114141930
H	2.40638675534796	5.74371089627877	12.75369503182559
N	0.47144833579872	8.44764722456431	12.46133643461210
N	0.13234332584472	11.09968587530766	12.72527879272219
S	2.20442922241242	12.67484531252017	14.67368299229179
S	3.10771266122266	7.67442183317112	13.98938966482813

UV-visible Absorption Spectroscopy

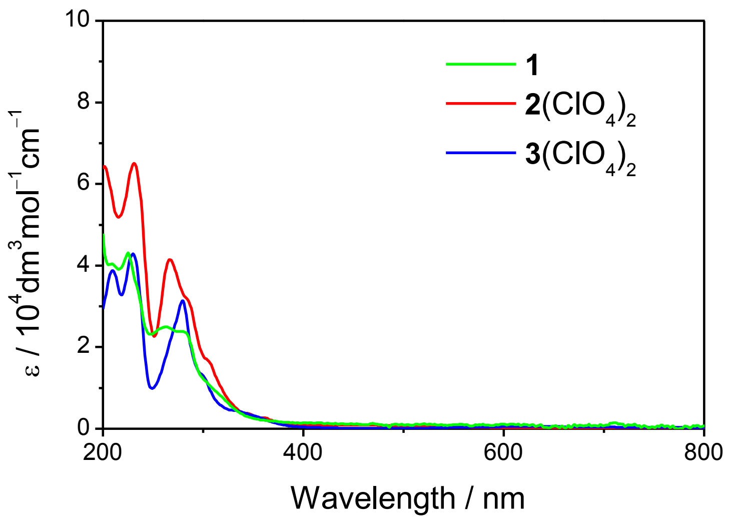

Figure S1. UV-visible absorption spectra of **1**, **2**$(ClO_4)_2$ and **3**$(ClO_4)_2$ in CH_3CN at 298 K; these absorption profiles remain unchanged for 16 h under ambient condition.

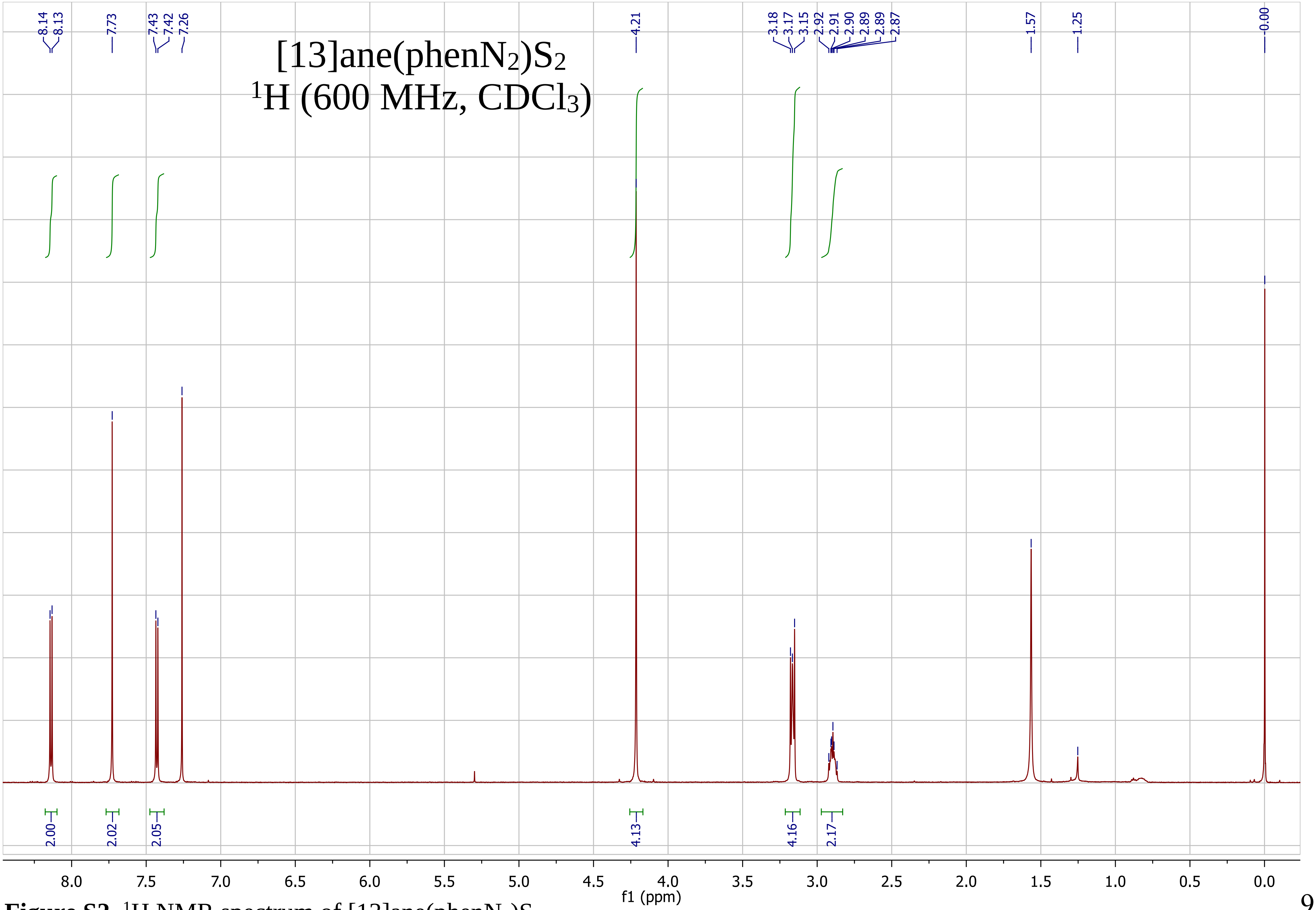

Figure S2. ^{1}H NMR spectrum of [13]ane(phenN_2)S_2.

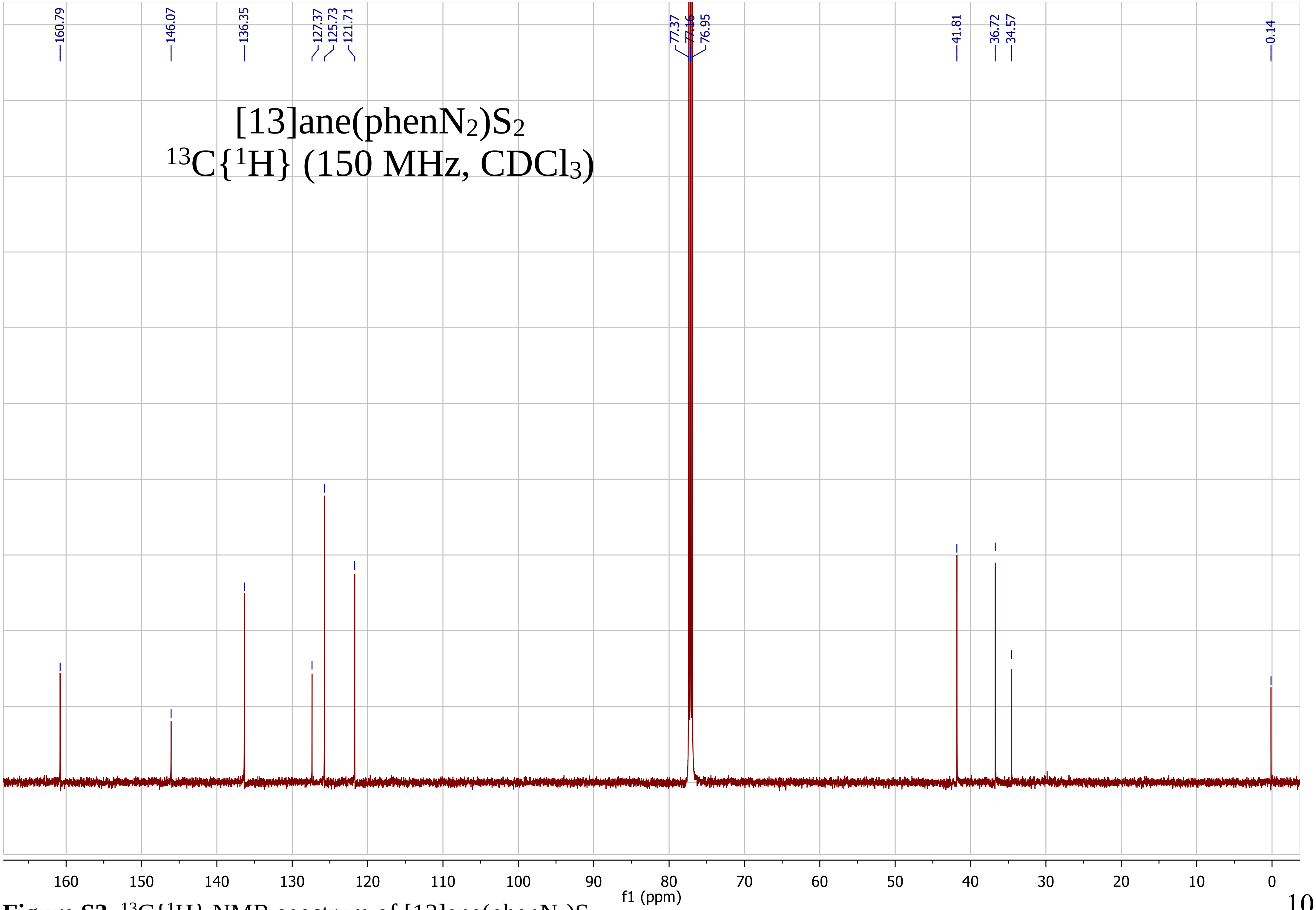

Figure S3. $^{13}C\{^{1}H\}$ NMR spectrum of [13]ane(phenN_2)S_2.

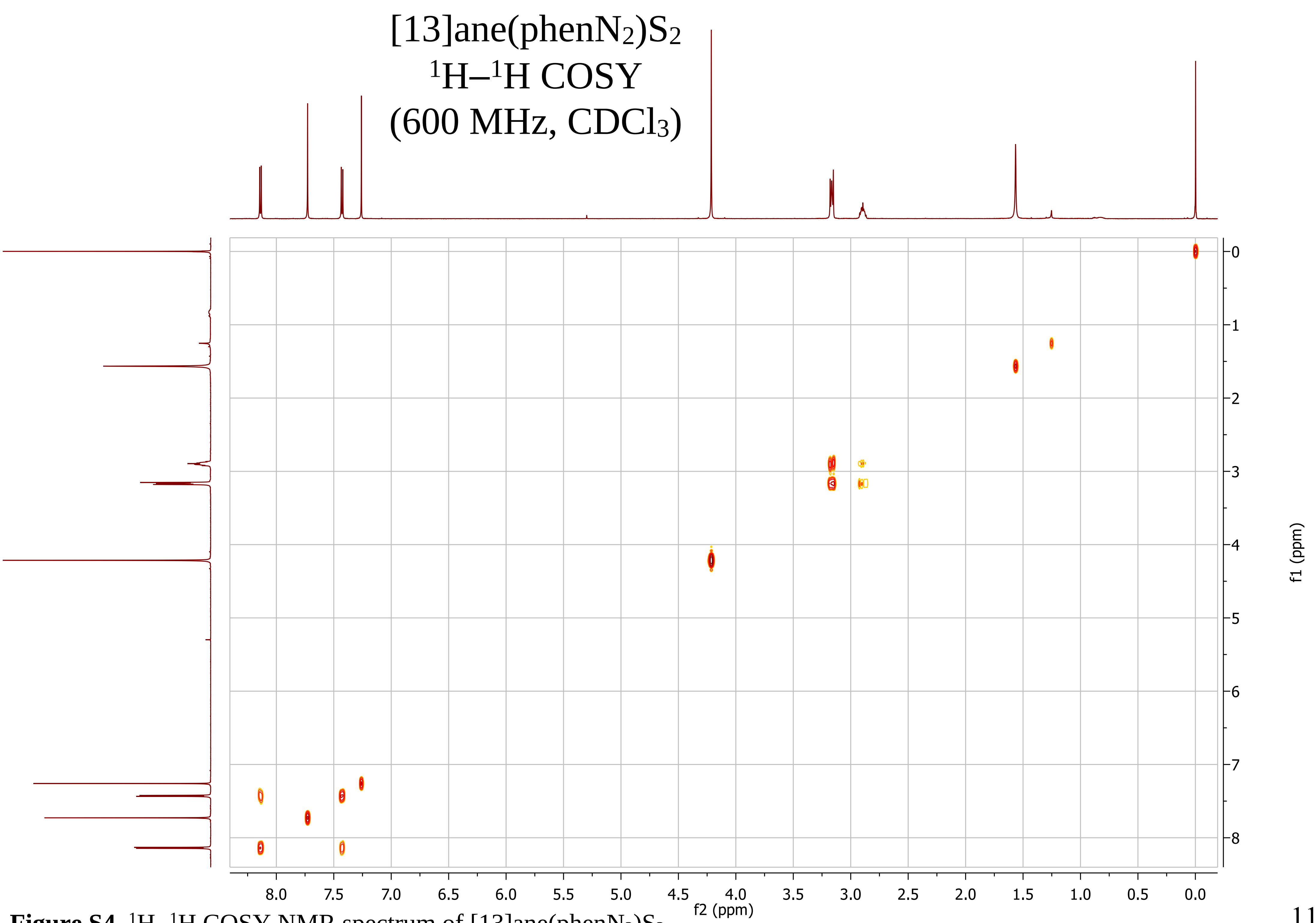

Figure S4. ^{1}H–^{1}H COSY NMR spectrum of [13]ane(phenN_2)S_2.

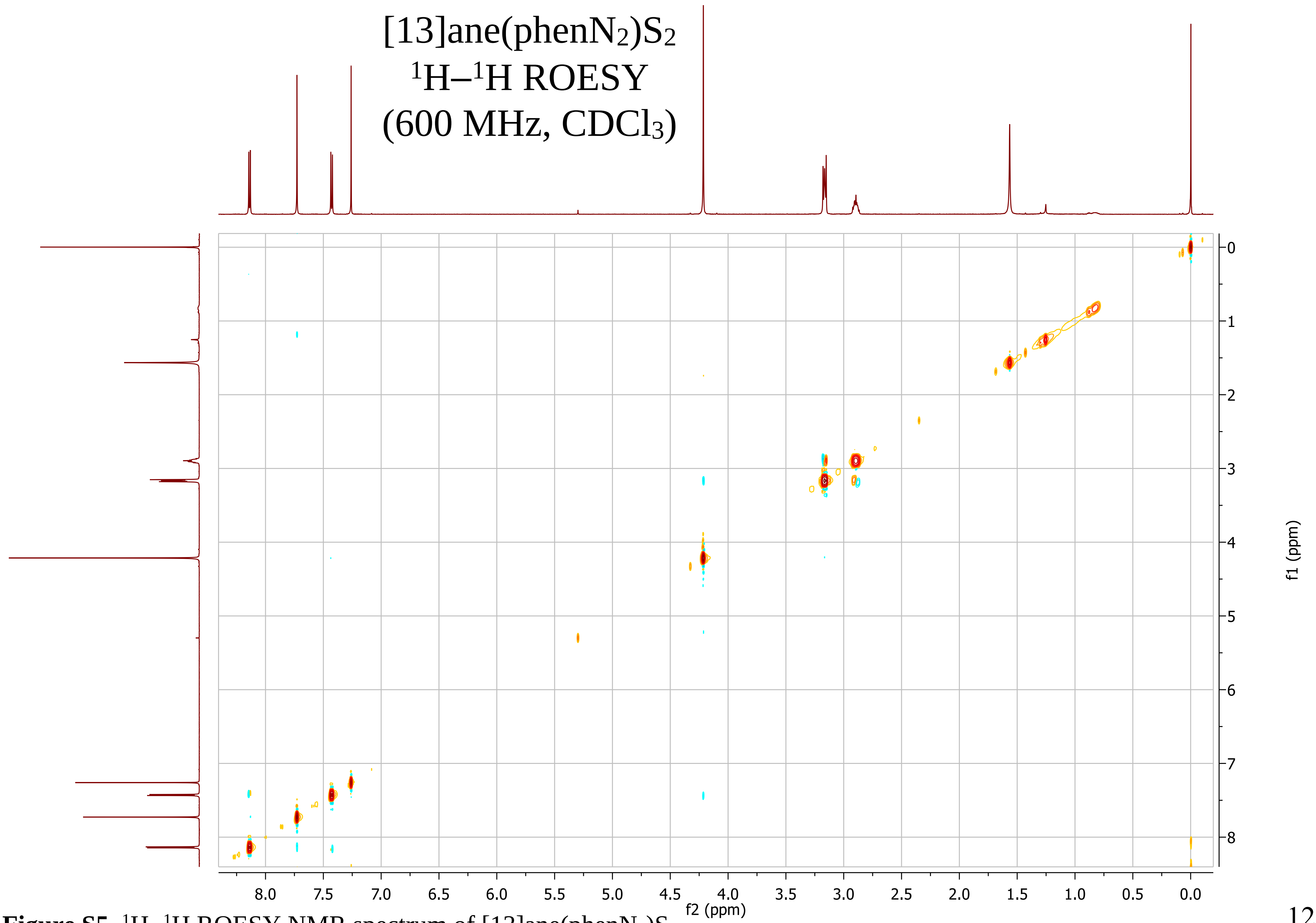

Figure S5. ^1H–^1H ROESY NMR spectrum of [13]ane(phenN$_2$)S$_2$.

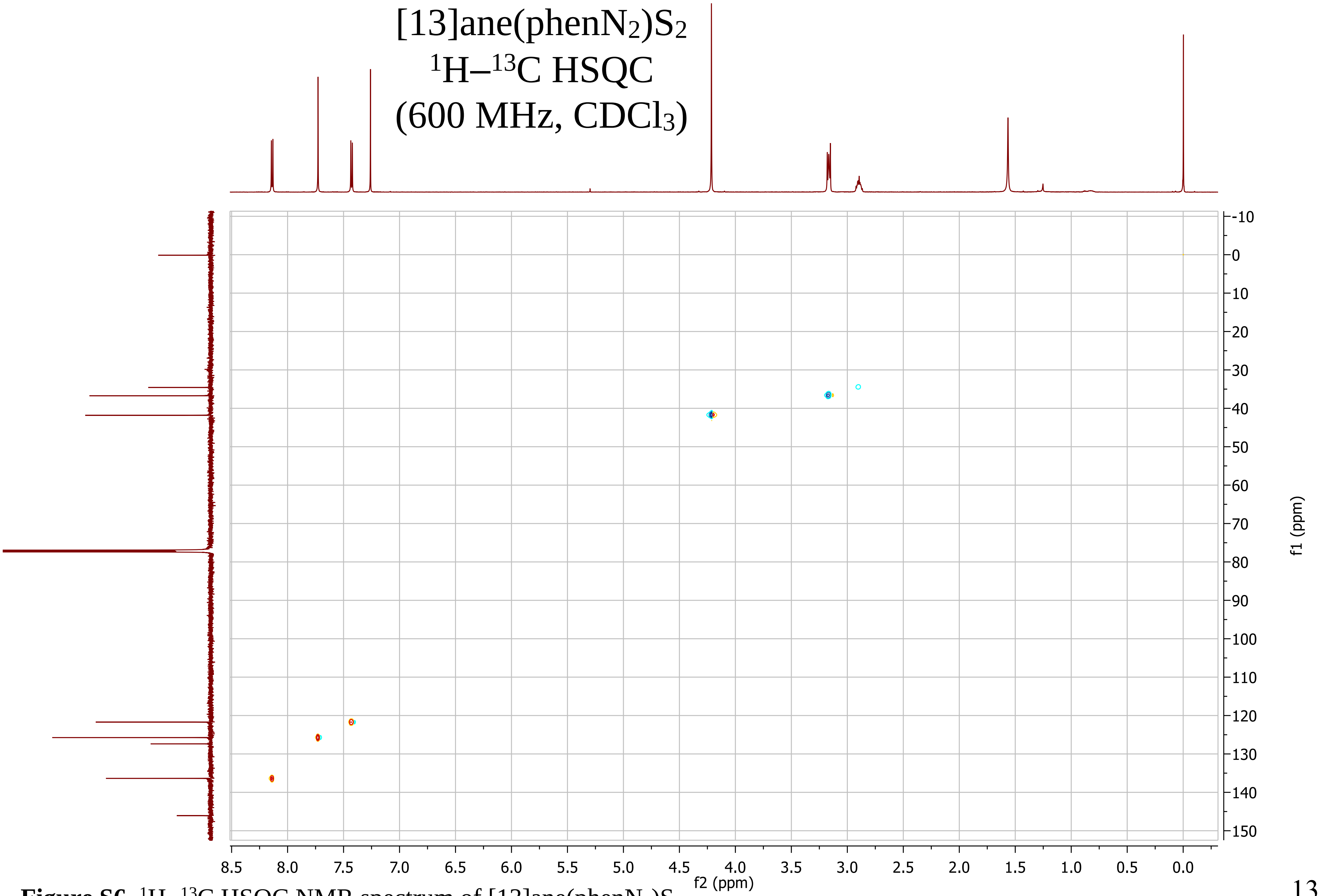

Figure S6. ^{1}H–^{13}C HSQC NMR spectrum of [13]ane(phenN_2)S_2.

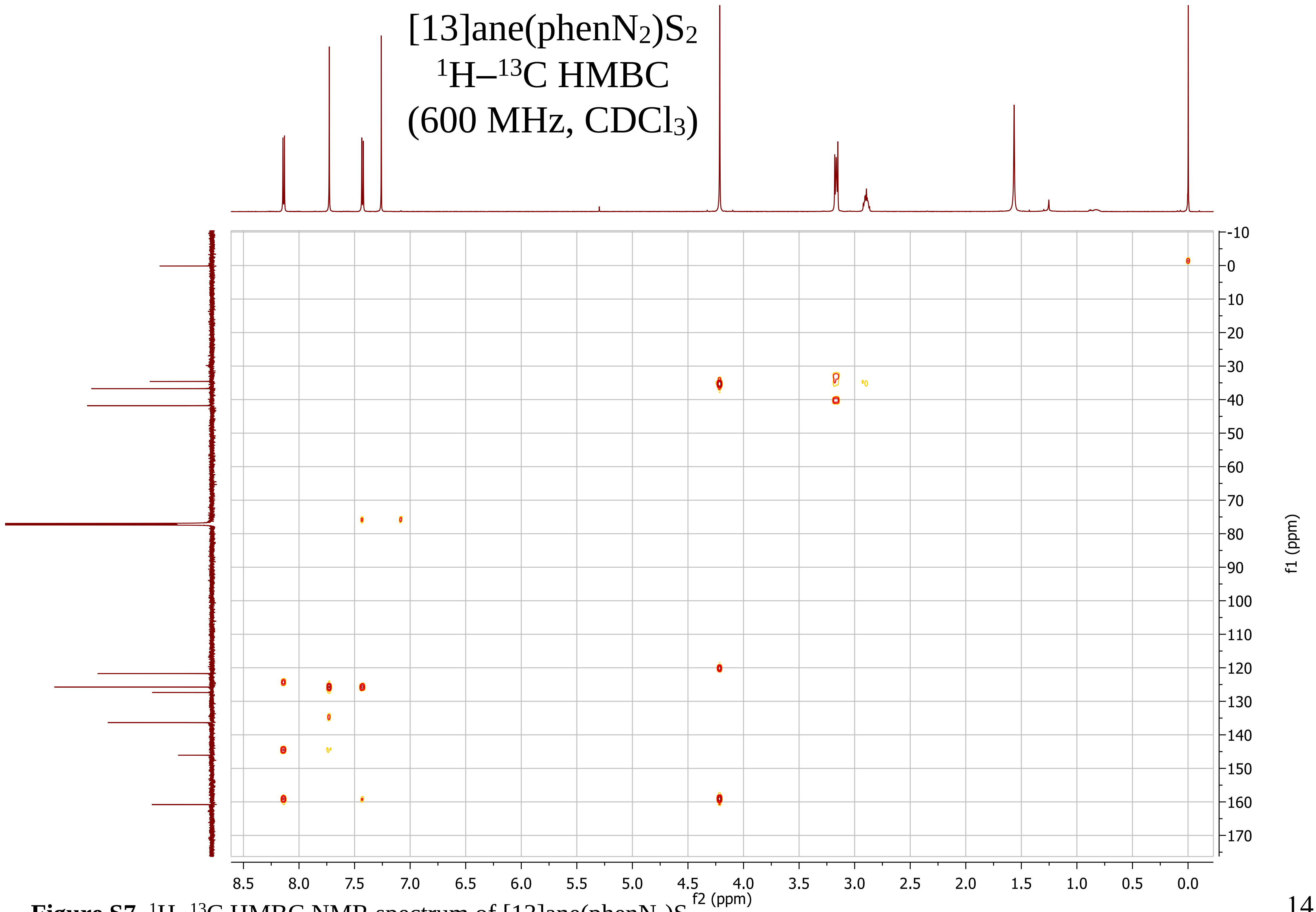

Figure S7. ^{1}H–^{13}C HMBC NMR spectrum of [13]ane(phenN_2)S_2.
